# Up‐regulation of LIMK1 expression in prostate cancer is correlated with poor pathological features, lymph node metastases and biochemical recurrence

**DOI:** 10.1111/jcmm.15138

**Published:** 2020-03-13

**Authors:** Jin‐Bei Huang, Yu‐Peng Wu, Yun‐Zhi Lin, Hai Cai, Shao‐Hao Chen, Xiong‐Lin Sun, Xiao‐Dong Li, Yong Wei, Qing‐Shui Zheng, Ning Xu, Xue‐Yi Xue

**Affiliations:** ^1^ Departments of Urology The First Affiliated Hospital of Fujian Medical University Fuzhou China

**Keywords:** advanced pathological features, biochemical recurrence, LIM domain kinase 1, lymph node metastasis, prostate cancer

## Abstract

This study aimed to explore the association between LIM domain kinase 1 (LIMK1) expression in prostate cancer (PCa) tissues with advanced pathological features, lymph node metastases and biochemical recurrence. A total of 279 PCa specimens from patients who underwent radical prostatectomy and 50 benign prostatic hyperplasia (BPH) specimens were collected to construct tissue microarray, which were subjected to immunohistochemical staining for LIMK1 expression subsequently. Logistic and Cox regression analysis were used to evaluate the relationship between LIMK1 expression and clinicopathological features of patients with PCa. Immunohistochemical staining assay demonstrated that LIMK1 expression was significantly higher in PCa than BPH specimens (77.1% vs 26.0%; *P* < .001). LIMK1 expression was significantly higher in positive lymph node specimens than corresponding PCa specimens (*P* = .002; *P* < .001). Up‐regulation of LIMK1 was associated with prostate volume, prostate‐specific antigen, prostate‐specific antigen density, Gleason score, T stage, lymph node metastases, extracapsular extension and seminal vesicle invasion, and positive surgical margin. Multivariate logistic regression analysis demonstrated that LIMK1 was an independent risk factor for PCa lymph node metastasis (*P* < .05). Multivariate Cox regression analysis revealed that the up‐regulation of LIMK1 was an independent risk factor for biochemical recurrence. Kaplan‐Meier analysis indicated that up‐regulation LIMK1 was associated with shortened biochemical‐free survival (BFS) after radical prostatectomy (*P* < .001). In conclusion, LIMK1 was significantly up‐regulated in PCa and positive lymph node specimens and correlated with lymph node metastasis and shortened BFS of PCa. The underlying molecular mechanism of LIMK1 in PCa should be further evaluated.

## 1 INTRODUCTION

Prostate cancer (PCa) is one of the most commonly diagnosed male malignancies and the second leading cause of cancer‐related death in men.^1^, ^2^ Unfortunately, most androgen‐dependent PCa (ADPC) inevitably progresses to castration‐resistant prostate cancer (CRPC) after androgen ablation therapy.^3^, ^4^ Metastasis is a complicated and multi‐step process. Invasion and distant metastasis are significantly associated with the prognosis of PCa. The prognosis was poor in patients with metastatic PCa because no curative treatment is currently available.

Co‐ordinated reorganization of the actin cytoskeleton is essential to tumour invasion and metastasis.^5^, ^6^, ^7^ LIM kinase 1 (LIMK1) is one of the members of the LIM kinase protein family.^6^, ^8^ Previous studies^6^, ^8^, ^9^, ^10^, ^11^ demonstrated that LIMK played an essential role in regulating the polymerization of actin through phosphorylation and inactivation of cofilin, which acted as the only downstream effector of LIMK1. Cofilin can be inactivated by LIMK1 when its Ser3 site was phosphorylated.^12^ Inactivated cofilin lost the ability of binding to actin filaments which improved the stability of F‐actin, resulting in the change of actin cytoskeleton.^13^ Several studies have now confirmed that the expression of LIMK1 is consistently elevated in the many kinds of tumours including breast cancer,^6^, ^14^ ovarian cancer,^15^, ^16^ colon cancer^17^, ^18^ and gastric cancer.^19^, ^20^ However, researchers have paid little attention to the role of LIMK1 in prostate cancer. Several studies have reported that LIMK can promote the invasive and metastatic ability of tumours.^8^, ^17^ What is more, it also participated in many kinds of biological behaviours including angiogenesis, proliferation, cell cycle and migration.^14^, ^21^, ^22^, ^23^ Thus, LIMK1 has great potential to be a therapeutic target to prevent the invasion and metastasis of PCa.

This study hypothesized that LIMK1 was high expression in PCa and was involved in the invasion and metastasis of PCa. The expression of LIMK1 in PCa was determined by immunohistochemistry, and the relationship between the expression of LIMK1 and the invasion, metastasis, and prognosis of PCa was analysed.

## 2 MATERIALS AND METHODS

### 2.1 Ethics

This study was approved by the Medical Ethics Committee of the First Affiliated Hospital of Fujian Medical University. Written informed consents were obtained from all patients.

### 2.2 Tissue specimen and data collection

A total of 279 specimens of PCa tissue were collected from the patients who underwent radical prostatectomy from January 2012 to September 2015. All specimens were pathologically confirmed as primary prostate adenocarcinoma. Patients without any preoperative endocrine therapy, chemotherapy, radiotherapy, and immunotherapy were included. The age of patients ranged from 48 to 78 years, with an average of 68.45 ± 6.92 years. The stage of PCa was classified based on the Union for International Cancer Control (UICC)‐TNM classification. Fifty benign prostatic hyperplasia specimens were treated as control.

### 2.3 Follow‐up

Patients with total prostate‐specific antigen (PSA) level less than 0.01 ng/mL were followed up for 1 month after radical prostatectomy. The follow‐up started from the date of the operation until the occurrence of the biochemical relapse. The follow‐up time ranged from 6 to 36 months. The exclusion criteria were as follows: patients who received adjuvant radiotherapy and/or endocrine therapy during follow‐up, with positive lymph node metastasis and with insufficient follow‐up data. Finally, a total of 163 patients were included in the analysis of biochemical recurrence. The follow‐up protocol^24^ was as follows: the first month after surgery; then every 3 months after surgery for 2 years; and afterwards every 6 months since the third year. The follow‐up was ended when biochemical recurrence occurred before September 2015. The total follow‐up time was 9 to 95 months, and the median follow‐up time was 55 months; the total biochemical recurrence rate was 17.18% (28/163), and the median biochemical recurrence time was 25 months (ranged from 10 to 67 months).

### 2.4 Construction of tissue microarray

The paraffin‐embedded benign prostatic hyperplasia and PCa specimens were obtained from the department of pathology of the First Affiliated Hospital of Fujian Medical University. The paraffin‐embedded specimens were sliced and underwent H&E staining. The representative areas of the H&E staining sections were evaluated and confirmed by a senior pathologist in order to construct tissue microarray. A tissue microarray maker was designed to generate tissue microarrays by using 2 × 2 mm tissue cores in each case. Finally, tissue microarrays contain 5 × 10 tissue cores for both PCa and benign prostatic hyperplasia specimens in each were obtained and then be sliced continuously into 4‐μm‐thick sections.

### 2.5 Immunohistochemistry

In the present study, staining of LIMK1 was performed by immunohistochemistry. Briefly, immunohistochemical staining for LIMK1 was performed on 4‐μm deparaffinized sections of formaldehyde‐fixed PCa and benign prostatic hyperplasia tissues using rabbit anti‐human LIMK1 polyclonal antibodies (Boster Biological Technology Co., Ltd.), and goat anti‐rabbit immunoglobulin G (Boster Biological Technology Co., Ltd.). Antibodies against LIMK1 were used in dilutions of 1:50. The sections for LIMK1 staining were treated with 0.01 mol/L citric acid buffer (PH 6.0l Fuzhou Maixin Biotech. Co., Ltd.) by a high‐pressure cooker 3 minutes for antigen retrieval.

The sections were then examined by light microscopy (Olympus) by two blinded pathologists. Any discrepancies were resolved by re‐reviewing the sections. Two semi‐quantitative methods and the total LIMK1 immunostaining score methods including staining intensity and the proportion of positive cells were described as follows.^25^, ^26^, ^27^, ^28^ The immunohistochemistry score of LIMK1 consists of two parts, including staining intensity and the proportion of positive cells. We classified the stating intensity as 0, absent; 1, weak; 2, moderate; and 3, strong. In terms of the proportion of positive cells, we defined the proportion as 0, <5%; 1, 5%‐25%; 2, 26%‐50%; 3, 51%‐75%; and 4, >75%. The immunohistochemistry score of LIMK1 was calculated utilizing the staining intensity score multiplied by the value of the percentage positivity score. The value of the LIMK1 immunohistochemistry score was ranged from 0 to 9. The expression level of LIMK1 was defined as ‘−’ (scores 0‐1), ‘+’ (scores 2‐3), ‘++’ (scores 4‐5) and ‘+++’ (scores ≥ 6).

### 2.6 Statistical methods

SPSS 21.0 statistical software (SPSS Inc) was used for all statistical analyses. The qualitative data were compared using the independent sample chi‐square test or Fisher exact test. The quantitative data were analysed using independent samples *t* test, Mann‐Whitney U test, Kruskal‐Wallis test or ANOVA. Kaplan‐Meier and the log‐rank test were used to compare the biochemical recurrence‐free survival in each group. *P* < .05 was considered statistically significant.

## 3 RESULTS

### 3.1 The different expression of LIMK1 between benign prostatic hyperplasia and PCa tissues

**Figure 1:**
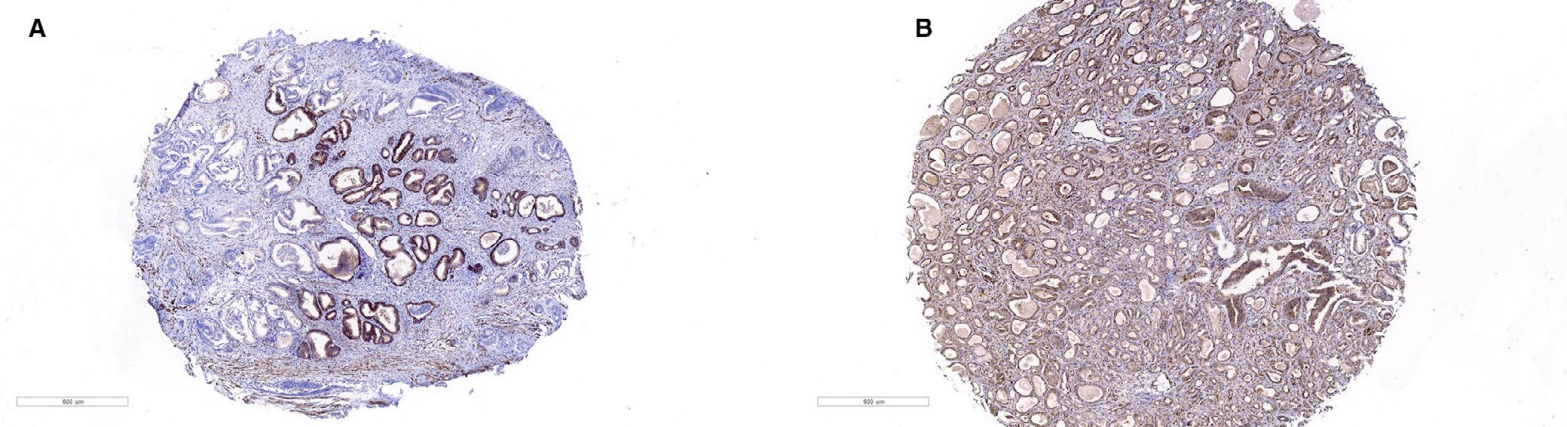
Tissue microarray containing normal prostate, benign prostate hyperplasia and prostate cancer tissues was immunostained with a monoclonal anti‐LIMK1 antibody. A, Benign prostatic hyperplasia tissues, 40×; B, prostate cancer tissue, 40×

A total of 215 cases of LIMK1‐positive expression were seen in 279 cases of PCa tissues, while a total 13 cases of LIMK1‐positive expression were observed in 50 cases of benign prostatic hyperplasia tissues. LIMK1 was mainly expressed in the cytoplasm of positive cells. The results demonstrated that the positive expression rate of LIMK1 in PCa tissues was significantly higher than that of benign prostatic hyperplasia tissues (77.1% vs 26.0%, respectively; *P* < .001; Table 1; Figure 1).

**Table 1:** The expression of LIMK1 in prostate cancer tissue and benign prostatic hyperplasia tissue

Group	LIMK1 expression				Positive rate (%)	*P* value
	−	+	++	+++		
BPH	37	10	3	0	26.0	<.001
Pca	64	68	73	74	77.1	

### 3.2 The different expression of LIMK1 between PCa tissues and corresponding lymph node metastases specimens

**Figure 2:**
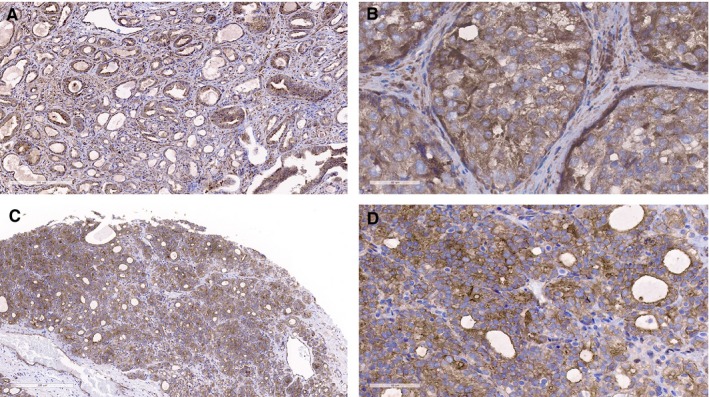
The expression of LIMK1 in positive lymph node metastasis tissue was higher than that in prostate cancer tissue. (A and B, prostate cancer tissue; C and D, lymph node metastasis; A and C, ×100; B and D, ×400)

A total of 40 cases of positive lymph node metastases specimens and corresponding PCa tissues were collected for immunohistochemistry. The results demonstrated that the positive expression of LIMK1 in lymph node metastases specimens was higher than that of corresponding PCa tissues (*P* = .002; Table 2; Figure 2).

**Table 2:** The expression of LIMK1 in prostate cancer and paired lymph node

Group	LIMK1 expression				*P* value
	−	+	++	+++	
Positive lymph node	0	5	8	27	.002
Prostate cancer	3	10	16	11	

### 3.3 The relationship between LIMK1 expression and clinicopathological features of PCa patients

**Figure 3:**
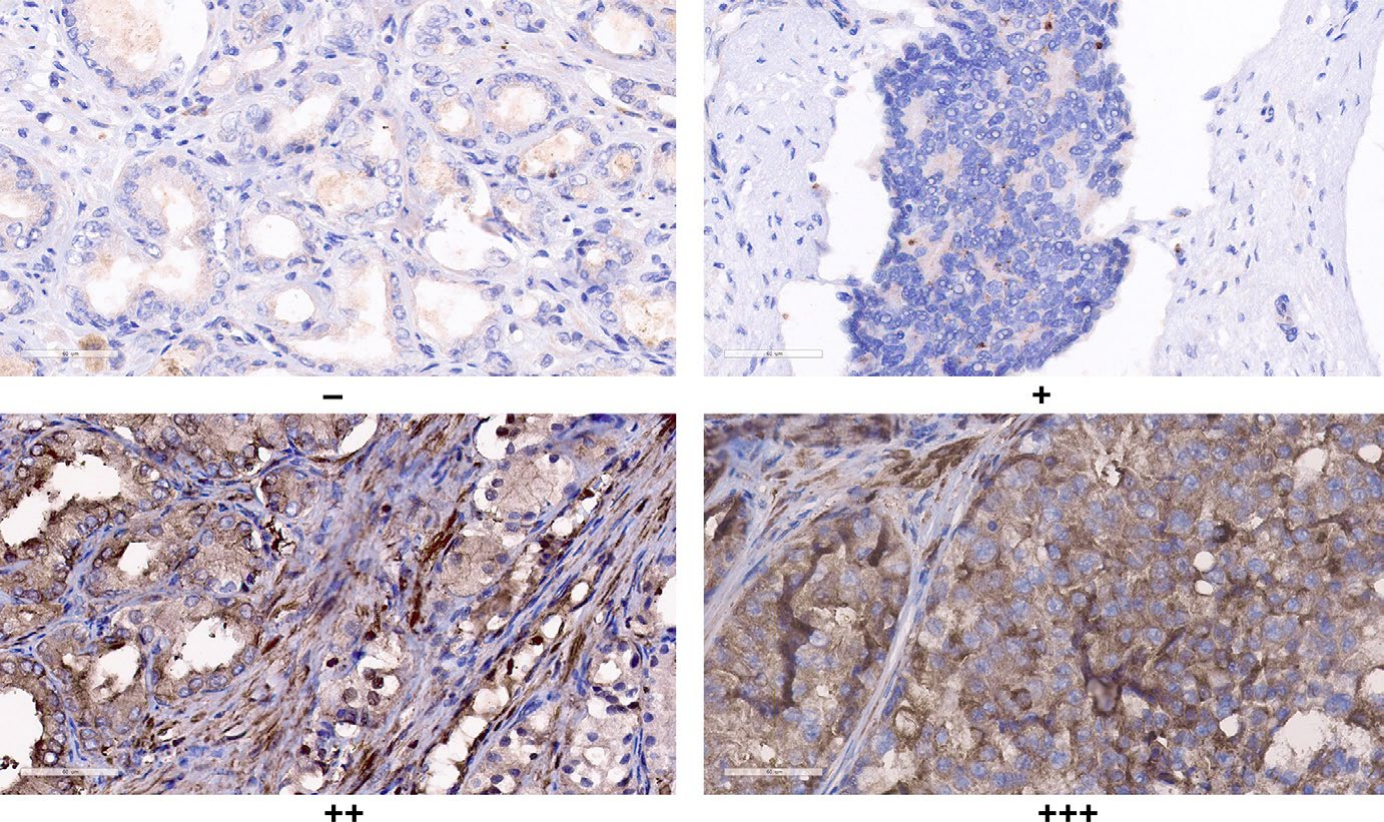
The expression of LIMK1 in prostate cancer. LIMK1 is strong staining in poorly differentiated, high stage and positive lymph node metastasis tissue (×400)

The expression of LIMK1 was significantly associated with the prostate volume, PSA level, PSA density, Gleason score, T stage, lymph node metastases, extracapsular extension and seminal vesicle invasion, and positive surgical margin (*P* < .05, Table 3). However, the expression of LIMK1 was not associated with the age of patients. Moreover, the strong staining of LIMK1 was seen in the low differentiation, high stage and lymph node metastasis specimens (Figure 3).

**Table 3:** Association of LIMK1 expression with clinicopathological features of prostate cancer

Variables	N	LIMK1 expression				*P* value
		−	+	++	+++	
Total, n (%)	279	64	68	73	74	
Age (years)						
<70	136 (48.7)	31 (48.4)	36 (52.9)	33 (45.2)	36 (48.6)	.839
≧70	143 (51.3)	33 (51.6)	32 (27.1)	40 (54.8)	38 (51.4)	
Prostate volume (cm^3^)						
≤35	99 (35.5)	33 (51.6)	43 (63.2)	15 (20.5)	8 (10.8)	<.001*
>35	180 (64.5)	31 (48.4)	25 (36.8)	58 (79.5)	66 (89.2)	
PSA (ng/mL)						
<10	42 (15.1)	12 (18.8)	18 (26.5)	6 (8.2)	6 (8.1)	.028*
10‐20	182 (65.2)	39 (60.9)	38 (55.9)	50 (68.5)	55 (74.3)	
>20	55 (19.7)	13 (20.3)	12 (17.6)	17 (23.3)	13 (17.6)	
PSAD (ng/mL·cm^3^)						
<0.15	18 (6.5)	2 (3.1)	11 (16.2)	2 (2.7)	3 (4.1)	.002*
≥0.15	261 (93.5)	62 (96.9)	57 (83.8)	71 (97.3)	71 (95.9)	
Gleason score						
2‐6	72 (25.8)	35 (54.7)	24 (35.3)	10 (13.7)	3 (4.1)	<.001*
7	141 (50.5)	19 (29.7)	33 (48.5)	39 (53.4)	50 (67.6)	
8‐10	66 (23.7)	10 (15.6)	11 (16.2)	24 (32.9)	21 (28.4)	
cT stage						
T1	42 (15.1)	28 (43.8)	6 (8.8)	4 (5.5)	4 (5.4)	<.001*
T2	192 (68.8)	31 (48.4)	60 (88.2)	56 (76.7)	45 (60.8)	
T3	45 (16.1)	5 (7.8)	2 (2.9)	13 (17.8)	25 (33.8)	
Lymph node metastasis						
Yes	95 (34.1)	4 (6.3)	17 (25.0)	32 (43.8)	42 (56.8)	<.001*
No	184 (65.9)	60 (93.8)	51 (75.0)	41 (56.2)	32 (43.2)	
Extracapsular extension						
Yes	45 (16.1)	5 (7.8)	2 (2.9)	13 (17.8)	25 (33.8)	<.001*
No	234 (83.9)	59 (92.2)	66 (97.1)	60 (82.2)	49 (66.2)	
Seminal vesicle invasion						
Yes	14 (5.0)	0 (0.0)	1 (1.5)	7 (9.6)	6 (8.1)	.016*
No	265 (95.0)	64 (100.0)	67 (98.5)	66 (90.4)	68 (91.9)	
Positive surgical margin						
Yes	33 (11.8)	6 (9.4)	3 (4.4)	9 (12.3)	15 (20.3)	.028*
No	246 (88.2)	58 (90.6)	65 (95.6)	64 (87.7)	59 (79.7)	

* *P* < .05.

Subsequently, subgroup analysis stratified by the lymph node metastasis was analysed. The results demonstrated that lymph node metastases were significantly associated with the preoperative PSA level, postoperative Gleason score, extracapsular extension, seminal vesicle invasion, positive surgical margin and the LIMK1 expression (*P* < .05, Table 4). However, lymph node metastases were not associated with the age, body mass index, prostate volume, the proportion of positive biopsy cores and PSA density (*P* > .05, Table 4).

**Table 4:** Association of clinicopathological features with lymph node metastasis of prostate cancer

Variables	N	Lymph node metastasis		*P* value
		Negative	Positive	
Total, n (%)	279	184	95	
Age (years)				
<70	136 (48.7)	91 (49.5)	45 (47.4)	.801
≧70	143 (51.3)	93 (50.5)	50 (52.6)	
BMI				
≤25	142 (50.9)	99 (53.8)	43 (45.3)	.207
>25	137 (49.1)	85 (46.2)	52 (54.7)	
Prostate volume (cm^3^)				
≤35	99 (35.5)	66 (35.9)	33 (34.7)	.895
>35	180 (64.5)	118 (64.1)	62 (65.3)	
Percentage of positive biopsies				
<50	176 (63.1)	117 (63.6)	59 (62.1)	.896
≥50	103 (36.9)	67 (36.4)	36 (37.9)	
PSA (ng/mL)				
<10	42 (15.1)	34 (18.5)	8 (8.4)	.015*
10‐20	182 (65.2)	121 (65.8)	61 (64.2)	
>20	55 (19.7)	29 (15.8)	26 (27.4)	
PSAD (ng/mL·cm^3^)				
<0.15	18 (6.5)	13 (7.1)	5 (5.3)	.619
≥0.15	261 (93.5)	171 (92.9)	90 (94.7)	
Gleason score				
2‐6	67 (36.4)	67 (36.4)	5 (5.3)	<.001*
7	80 (43.5)	80 (43.5)	61 (64.2)	
8‐10	66 (20.1)	37 (20.1)	29 (30.5)	
cT stage				
T1	42 (15.1)	37 (20.1)	5 (5.3)	.001*
T2	192 (68.8)	124 (67.4)	68 (71.5)	
T3	45 (16.1)	23 (12.5)	22 (23.2)	
Extracapsular extension				
Yes	234 (83.9)	161 (87.5)	73 (76.8)	.026*
No	45 (16.1)	23 (12.5)	22 (23.2)	
Seminal vesicle invasion				
Yes	265 (95.0)	179 (97.3)	86 (90.5)	.020*
No	14 (5.0)	5 (2.7)	9 (9.5)	
Positive surgical margin				
Yes	246 (88.2)	169 (91.8)	77 (81.1)	.011*
No	33 (11.8)	15 (8.2)	18 (18.9)	
LIMK1 expression				
−	64 (22.9)	60 (32.6)	4 (4.2)	<.001*
+	68 (24.4)	51 (27.7)	17 (17.9)	
++	73 (26.2)	41 (22.3)	32 (33.7)	
+++	74 (26.5)	32 (17.4)	42 (44.2)	

* *P* < .05.

The multivariate logistic regression analysis demonstrated that LIMK1 was independent risk factor for PCa lymph node metastasis (*P* < .001, Table 5).

**Table 5:** Logistic regression analysis of influencing factors for prostate cancer lymph node metastasis

Variable	OR (95% CI)	*P* value
PSA (ng/mL, <10 vs 10‐20 vs >20)	1.653 (0.892‐3.065)	.111
Gleason score (2‐6 vs 7 vs 8‐10)	1.626 (0.970‐2.725)	.065
T stage (T1 vs T2 vs T3)	1.120 (0.338‐3.713)	.853
Extracapsular extension	0.443 (0.098‐1.997)	.289
Seminal vesicle invasion	1.469 (0.312‐6.921)	.626
Positive surgical margin	1.379 (0.428‐4.440)	.590
LIMK1 expression (−/+/++/+++)	2.289 (1.694‐3.092)	<.001*

* *P* < .05.

### 3.4 The relationship between LIMK1 expression and biochemical recurrence

Univariate Cox regression analysis demonstrated that higher proportion of positive biopsy cores, T stage, Gleason score, extracapsular extension, positive surgical margin and LIMK1 expression was associated with biochemical recurrence (*P* < .05, Table 6). Multivariate Cox regression analysis revealed that up‐regulation of LIMK1 was independent risk factor for biochemical recurrence (*P* < .05, Table 6).

**Table 6:** Univariate and multivariate analysis of risk factors for biochemical recurrence

Variable	Univariate		Multivariate	
	HR (95% CI)	*P* value	HR (95% CI)	*P* value
Age (years, <70 vs ≥70)	1.593 (0.735‐3.451)	.238		
BMI (kg/m^2^, ≤25 vs >25)	1.745 (0.825‐3.694)	.145		
Prostate volume (cm^3^, ≤35 vs >35)	1.494 (0.658‐3.393)	.337		
PSA (ng/mL, <10 vs 10‐20 vs >20)	1.185 (0.633‐2.220)	.595		
PSAD (ng/mL·cm^3^, <0.15 vs ≥0.15)	1.856 (0.252‐13.675)	.544		
Percentage of positive biopsies (%, <50 vs ≥50)	0.347 (0.132‐0.914)	.032*	0.523 (0.132‐2.079)	.357
T stage (T1 vs T2 vs T3)	2.798 (1.434‐5.460)	.003*	0.295 (0.045‐1.947)	.205
Gleason score (2‐6 vs 7 vs 8‐10)	1.928 (1.170‐3.175)	.010*	1.189 (0.514‐2.755)	.686
Extracapsular extension (Yes vs No)	3.818 (1.679‐8.685)	.001*	7.796 (0.908‐66.921)	.061
Seminal vesicle invasion (Yes vs No)	1.293(0.176‐9.523)	.801		
Positive surgical margin (Yes vs No）	4.188 (1.693‐10.358)	.002*	1.068 (0.314‐3.640)	.916
LIMK1 expression (−/+/++/+++)	3.020 (2.004‐4.549)	<.001*	2.933 (1.118‐3.724)	<.001*

* *P* < .05.

**Figure 4:**
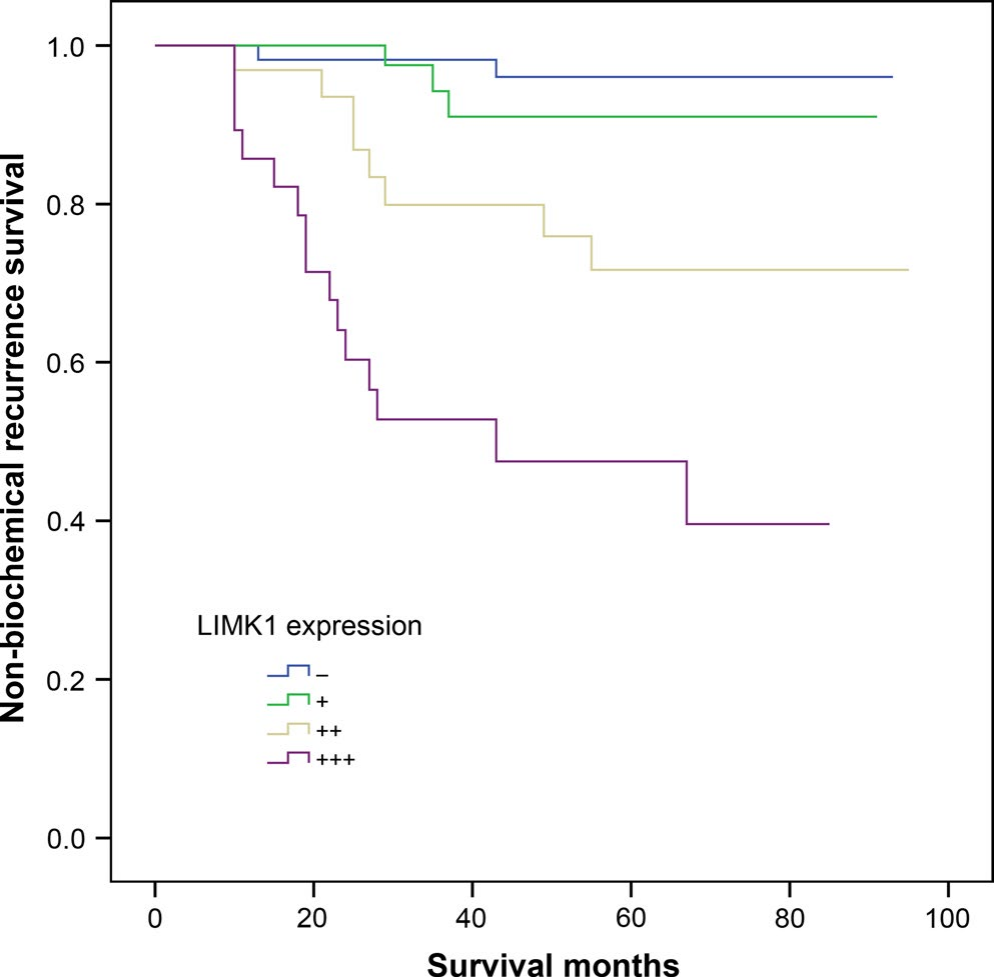
Non‐biochemical recurrence survival in patients with prostate cancer stratified by the different expression level of LIMK1 protein

Kaplan‐Meier analysis indicated that up‐regulation LIMK1 was associated with shortened biochemical‐free survival (BFS) after radical prostatectomy (*P* < .001, Figure 4).

## 4 DISCUSSION

The LIMK family consists of LIMK1 and LIMK2, which belongs to the serine protein kinase, and associated with actin polymerization and microtubule depolymerization.^29^ The expression of LIMK1 was mainly seen in the cytoplasm and can freely shuttle between normal nucleus and cytoplasm.^9^ The expression of LIMK was elevated in many kinds of tumours, especially highly invasive malignancies. LIMK 1 plays an important role in the invasion and metastases of tumours by regulating the actin cytoskeleton molecules.^14^, ^15^, ^17^ Recently, the significance of LIMK1 in tumorigenesis has aroused extensive concern.^6^, ^19^ There are many mechanisms regulating the activation of LIMKI. The activated LIMK1 is responsible for the stability of the cytoskeleton and the bond of external stimulation of the cells.^9^ The LIMK1 was in the cytoplasm and rapidly migrates back and forth between the nucleus and the cytoplasm.^11^, ^29^ When the cytoskeleton is assembled, LIMK1 deactivates the cofilin by phosphorylation of 3 serine residues, which reverses the process of actin depolymerization.^8^ It is reported that LIMK1 played an important role in regulating the transportation process of lysosome and endosome.^6^ In addition, Manetti et al^30^ also demonstrated that metastasis‐related gene is located on chromosome 7q11.2 and LIMK1 is also located on chromosome 7q11.2. These results indicated that LIMK1 may play an important role in tumour metastasis.

Lymph node metastasis frequently occurs in PCa, especially pelvic lymph node metastasis, which plays an important role in poor prognosis of PCa and is the key step of systemic metastasis.^31^ Nowadays, it is still controversial that pelvic lymph node dissection should be performed with radical prostatectomy. Some researchers^32^, ^33^ suggest that pelvic lymph node dissection can increase the accuracy of tumour stage and improve the prognosis of patients in some extent. However, for patients with pathological confirmed pTxN0 PCa, the lymph node dissection does not improve the prognosis, even in patients with high PSA level, high pathological stage, and extracapsular extension prior to the surgery.^34^, ^35^ So far, researchers suggested that patients with low‐risk PCa should avoid pelvic lymph node dissection, while patients with moderate‐risk or high‐risk PCa, especially high‐risk PCa, are recommended to underwent standard or extended pelvic lymph node dissection.^36^, ^37^ In general, however, there is still a lack of an effective way to predict the risk of lymph node metastasis preoperatively and evaluate the benefit obtained from the lymph node dissection. In this study, the expression of LIMK1 in positive lymph nodes and the corresponding primary PCa tissues was evaluated. The results demonstrated that the expression of LIMK1 in positive lymph nodes was higher than that of the corresponding primary lesions. Subgroup analysis stratified by the lymph node metastasis demonstrated that lymph node metastases were significantly associated with the preoperative PSA level, postoperative Gleason score, extracapsular extension, seminal vesicle invasion, positive surgical margin and the LIMK1 expression. Moreover, the multivariate logistic regression analysis demonstrated that LIMK1 was independent risk factor for PCa lymph node metastasis. It is indicated that the expression of the LIMK1 could be treated as a predictor of the invasion and metastasis of PCa.

At present, there are many clinicopathological parameters to evaluate the risk of progression, metastasis and prognosis of PCa. The risk factors for biochemical recurrence included body mass index, preoperative PSA, the proportion of positive biopsy cores, pathological stage, extracapsular extension, seminal vesical invasion, lymph node metastasis, and positive surgical margin.^38^, ^39^, ^40^ However, the accuracy of these traditional clinicopathological factors for predicting the biochemical recurrence of PCa remains low.^41^ There is still no ideal and reliable marker for predicting the tumour growth, invasion and metastasis.^42^ Sen et al^43^ reported that the serum LIMK1 level in patients with hepatocellular carcinoma was significantly higher than those in patients with liver cirrhosis and normal individuals. The diagnostic accuracy of LIMK1 in the diagnosis of hepatocellular carcinoma was higher than that of AFP. In the study of cytotoxic chemotherapy of triple‐negative breast cancer, the expression of LIMK1 was associated with the prognosis of the cytotoxic chemotherapy.^44^ Manevich et al^45^ indicated that the expression of LIMK1 in PCa tissues was increased when compared with the adjacent PCa tissues. The elevated LIMK1 expression was also correlated with the occurrence of castration‐resistant PCa after surgery. The expression of LIMK1 increased in a short time was correlated with an increased risk of bone metastasis of PCa. Multivariate Cox regression analysis revealed that up‐regulation of LIMK1 was independent risk factor for biochemical recurrence. Kaplan‐Meier analysis indicated that up‐regulation LIMK1 was associated with shortened biochemical‐free survival (BFS) after radical prostatectomy. These results indicated that LIMK1 is an ideal and reliable biomarker to predict the risk of biochemical recurrence. For patients with an elevated expression of LIMK1, early use of adjuvant radiotherapy or endocrine therapy may postpone the occurrence of biochemical recurrence.

There are some limitations in this study. Firstly, this study was retrospectively designed. Secondly, the prognostic power of the number of positive lymph nodes with that of lymph node ratio was not analysed in this study.

In conclusion, LIMK1 was significantly up‐regulated in PCa and positive lymph node specimens and correlated with lymph node metastasis and shortened BFS of PCa. The underlying molecular mechanism of LIMK1 in PCa should be further evaluated.

## CONFLICT OF INTEREST

The authors declare that they have no competing interests.

## AUTHOR CONTRIBUTIONS

NX, XYX and JBH conceived and designed the experiments; YPW and YZL performed the experiments; SHC, XLS and XDL analysed the data; NX, XYX, YW and QSZ contributed reagents/materials/analysis tools; and YPW, JBH, YZL and HC wrote the paper. All authors read and approved the final manuscript.

## Data Availability

All data generated or analysed during this study are included in this article.

## References

[1] Jiao LI , Li Y , Shen D , et al. The prostate cancer‐up‐regulated Myc‐associated zinc‐finger protein (MAZ) modulates proliferation and metastasis through reciprocal regulation of androgen receptor. Med Oncol. 2013;30:1‐8. 10.1007/s12032-013-0570-3 23609189

[2] Xu N , Wu YP , Ke ZB , et al. Identification of key DNA methylation‐driven genes in prostate adenocarcinoma: an integrative analysis of TCGA methylation data. J Transl Med. 2019;17(1):311. 31533842 10.1186/s12967-019-2065-2 PMC6751626

[3] Xu B , Wang N , Wang X , et al. MiR‐146a suppresses tumor growth and progression by targeting EGFR pathway and in a p‐ERK‐dependent manner in castration‐resistant prostate cancer † ‡. Prostate. 2012;72:1171‐1178. 22161865 10.1002/pros.22466

[4] Cornford P , Bellmunt J , Bolla M , et al. EAU guidelines on prostate cancer. Part II: treatment of relapsing, metastatic, and castration‐resistant prostate cancer. Eur Urol. 2016;35:565. 10.1016/j.eururo.2016.08.002 27591931

[5] Shankar J , Nabi IR . Actin cytoskeleton regulation of epithelial mesenchymal transition in metastatic cancer cells. PloS One. 2015;10(7):e0132759. 25756282 10.1371/journal.pone.0119954 PMC4355409

[6] Mcconnell BV , Koto K , Gutierrezhartmann A . Nuclear and cytoplasmic LIMK1 enhances human breast cancer progression. Mol Cancer. 2011;10:75. 21682918 10.1186/1476-4598-10-75 PMC3131252

[7] Peckham M . How myosin organization of the actin cytoskeleton contributes to the cancer phenotype. Biochem Soc Trans. 2016;44:1026. 27528748 10.1042/BST20160034

[8] Zhou Y , Su J , Shi L , Liao Q , Su Q . DADS downregulates the Rac1‐ROCK1/PAK1‐LIMK1‐ADF/cofilin signaling pathway, inhibiting cell migration and invasion. Oncol Rep. 2013;29:605‐612. 23233092 10.3892/or.2012.2168

[9] Estornes Y , Gay F , Gevrey JC , et al. Differential involvement of destrin and cofilin‐1 in the control of invasive properties of Isreco1 human colon cancer cells. Int J Cancer. 2007;121:2162‐2171. 17583572 10.1002/ijc.22911

[10] Hotulainen P , Paunola E , Vartiainen MK , Lappalainen P . Actin‐depolymerizing factor and cofilin‐1 play overlapping roles in promoting rapid F‐actin depolymerization in mammalian nonmuscle cells. Mol Biol Cell. 2005;16:649‐664. 15548599 10.1091/mbc.E04-07-0555 PMC545901

[11] Li Y , Hu F , Chen HJ , et al. LIMK‐dependent actin polymerization in primary sensory neurons promotes the development of inflammatory heat hyperalgesia in rats. Sci Signal. 2014;7:ra61. 24962708 10.1126/scisignal.2005353

[12] Heredia L , Helguera P , de Olmos OS , et al. Phosphorylation of actin‐depolymerizing factor/cofilin by LIM‐kinase mediates amyloid beta‐induced degeneration: a potential mechanism of neuronal dystrophy in Alzheimer's disease. J Neurosci. 2006;26:6533‐6542. 16775141 10.1523/JNEUROSCI.5567-05.2006 PMC6674046

[13] Grintsevich EE , Reisler E . Drebrin inhibits cofilin‐induced severing of F‐actin. Cytoskeleton. 2014;71:472‐483. 25047716 10.1002/cm.21184 PMC4465285

[14] Li D , Song H , Wu T , et al. MiR‐138‐5p targeting LIMK1 suppresses breast cancer cell proliferation and motility. RSC Adv. 2017;7:52030‐52038.

[15] Chen P , Zeng M , Zhao Y , Fang X . Upregulation of Limk1 caused by microRNA‐138 loss aggravates the metastasis of ovarian cancer by activation of Limk1/cofilin signaling. Oncol Rep. 2014;32:2070‐2076. 25190487 10.3892/or.2014.3461

[16] Zhang W , Gan N , Zhou J . Immunohistochemical investigation of the correlation between LIM kinase 1 expression and development and progression of human ovarian carcinoma. J Int Med Res. 2012;40:1067‐1073. 22906279 10.1177/147323001204000325

[17] Jian S , Zhou Y , Pan Z , et al. Downregulation of LIMK1–ADF/cofilin by DADS inhibits the migration and invasion of colon cancer. Sci Rep. 2017;7:45624. 28358024 10.1038/srep45624 PMC5372356

[18] Liao Q , Rui L , Rui Z , et al. LIM kinase 1 interacts with myosin‐9 and alpha‐actinin‐4 and promotes colorectal cancer progression. Br J Cancer. 2017;117(4):563‐571. 28664914 10.1038/bjc.2017.193 PMC5558682

[19] Li X , Ke Q , Li Y , Liu F , Zhu G , Li F . DGCR6L, a novel PAK4 interaction protein, regulates PAK4‐mediated migration of human gastric cancer cell via LIMK1. Int J Biochem Cell Biol. 2010;42:70‐79. 19778628 10.1016/j.biocel.2009.09.008

[20] You T , Gao W , Wei J , et al. Overexpression of LIMK1 promotes tumor growth and metastasis in gastric cancer. Biomed Pharmacother. 2015;69:96‐101. 25661344 10.1016/j.biopha.2014.11.011

[21] Vlecken DH , Bagowski CP . LIMK1 and LIMK2 are important for metastatic behavior and tumor cell‐induced angiogenesis of pancreatic cancer cells. Zebrafish. 2009;6:433‐439. 20047470 10.1089/zeb.2009.0602

[22] Wan L , Zhang L , Fan K , Wang J . MiR‐27b targets LIMK1 to inhibit growth and invasion of NSCLC cells. Mol Cell Biochem. 2014;390:85‐91. 24390089 10.1007/s11010-013-1959-1

[23] Sumi T , Hashigasako A , Matsumoto K , Nakamura T . Different activity regulation and subcellular localization of LIMK1 and LIMK2 during cell cycle transition. Exp Cell Res. 2006;312:1021‐1030. 16455074 10.1016/j.yexcr.2005.12.030

[24] Hong X , Sin WC , Harris AL , Naus CC . Gap junctions modulate glioma invasion by direct transfer of microRNA. Oncotarget. 2015;6:15566‐15577. 25978028 10.18632/oncotarget.3904 PMC4558171

[25] Sakamoto S , Mccann RO , Dhir R , Kyprianou N . Talin1 promotes tumor invasion and metastasis via focal adhesion signaling and anoikis resistance. Cancer Res. 2010;70:1885‐1895. 20160039 10.1158/0008-5472.CAN-09-2833 PMC2836205

[26] Budwit‐Novotny DA , Mccarty KS , Cox EB , et al. Immunohistochemical analyses of estrogen receptor in endometrial adenocarcinoma using a monoclonal antibody. Cancer Res. 1986;46:5419‐5425. 3756890

[27] Ma HQ , Liang XT , Zhao JJ , et al. Decreased expression of Neurensin‐2 correlates with poor prognosis in hepatocellular carcinoma. World J Gastroenterol. 2009;15:4844‐4848. 19824122 10.3748/wjg.15.4844 PMC2761566

[28] Zheng QS , Chen SH , Wu YP , et al. Increased Paxillin expression in prostate cancer is associated with advanced pathological features, lymph node metastases and biochemical recurrence. J Cancer. 2018;9(6):959‐967. 29581775 10.7150/jca.22787 PMC5868163

[29] Scott RW , Olson MF . LIM kinases: function, regulation and association with human disease. J Mol Med. 2007;85:555‐568. 17294230 10.1007/s00109-007-0165-6

[30] Manetti F . LIM kinases are attractive targets with many macromolecular partners and only a few small molecule regulators †. Med Res Rev. 2012;32:968‐998. 22886629 10.1002/med.20230

[31] Verhagen PC , Schröder FH , Collette L , Bangma CH . Does local treatment of the prostate in advanced and/or lymph node metastatic disease improve efficacy of androgen‐deprivation therapy? A systematic review. Eur Urol. 2010;58:261‐269. 20627403 10.1016/j.eururo.2010.05.027

[32] Abdollah F , Gandaglia G , Suardi N , et al. More extensive pelvic lymph node dissection improves survival in patients with node‐positive prostate cancer. Eur Urol. 2015;68:e35. 24882672 10.1016/j.eururo.2014.05.011

[33] Moschini M , Briganti A , Murphy CR , et al. Outcomes for patients with clinical lymphadenopathy treated with radical prostatectomy. Eur Urol. 2016;69:193‐196. 26264160 10.1016/j.eururo.2015.07.047

[34] Dimarco DS , Zincke H , Sebo TJ , Slezak J , Bergstralh EJ , Blute ML . The extent of lymphadenectomy for pTXNO prostate cancer does not affect prostate cancer outcome in the prostate specific antigen era. J Urol. 2005;173:1121‐1125. 15758719 10.1097/01.ju.0000155533.93528.4c

[35] Murphy AM , Berkman DS , Desai M , Benson MC , Mckiernan JM , Badani KK . The number of negative pelvic lymph nodes removed does not affect the risk of biochemical failure after radical prostatectomy. BJU Int. 2010;105:176‐179. 19549117 10.1111/j.1464-410X.2009.08707.x PMC5508720

[36] Ji J , Yuan H , Wang L , Hou J . Retraction: “Is the impact of the extent of lymphadenectomy in radical prostatectomy related to the disease risk? A single center prospective study” J Surg Res 2012;178:779–784. J Surg Res. 2012;178:779‐784. 22819313 10.1016/j.jss.2012.06.069

[37] Schiavina R , Manferrari F , Garofalo M , et al. The extent of pelvic lymph node dissection correlates with the biochemical recurrence rate in patients with intermediate‐ and high‐risk prostate cancer. Eur Urol Suppl. 2011;10:1262‐1268. 10.1111/j.1464-410X.2010.10016.x 21446934

[38] Deng FM , Donin N , Benito RP , et al. Size‐adjusted quantitative gleason score as a predictor of biochemical recurrence after radical prostatectomy. Eur Urol. 2015;70:248‐253. 26525839 10.1016/j.eururo.2015.10.026 PMC4963258

[39] Bai PD , Hu MB , Xu H , et al. Body mass index is associated with higher Gleason score and biochemical recurrence risk following radical prostatectomy in Chinese men: a retrospective cohort study and meta‐analysis. World J Surg Oncol. 2015;13:311. 26542246 10.1186/s12957-015-0725-0 PMC4635546

[40] Kang HW , Do JH , Yong LJ , et al. Prostate‐specific antigen density predicts favorable pathology and biochemical recurrence in patients with intermediate‐risk prostate cancer. Asian J Androl. 2016;18:480‐484. 26178393 10.4103/1008-682X.154313 PMC4854109

[41] Capitanio U , Briganti A , Gallina A , et al. Predictive models before and after radical prostatectomy. Prostate. 2010;70:1371‐1378. 20623635 10.1002/pros.21159

[42] Heidenreich A , Bellmunt J , Bolla M , et al. EAU guidelines on prostate cancer. Part 1: screening, diagnosis, and treatment of clinically localised disease. Eur Urol. 2011;59:61‐71. 21056534 10.1016/j.eururo.2010.10.039

[43] Sen S , Ng WP , Kumar S . Contributions of talin‐1 to glioma cell‐matrix tensional homeostasis. J R Soc Interface. 2012;9:1311‐1317. 22158841 10.1098/rsif.2011.0567 PMC3350720

[44] Prunier C , Josserand V , Vollaire J , et al. LIM kinase inhibitor Pyr1 reduces the growth and metastatic load of breast cancers. Cancer Res. 2016;76:3541‐3552. 27216191 10.1158/0008-5472.CAN-15-1864

[45] Manevich E , Grabovsky V , Feigelson SW , Alon R . Talin 1 and paxillin facilitate distinct steps in rapid VLA‐4‐mediated adhesion strengthening to vascular cell adhesion molecule 1. J Biol Chem. 2007;282:25338‐25348. 17597073 10.1074/jbc.M700089200

